# Superior vena cava syndrome in a patient with locally advanced lung cancer with good response to definitive chemoradiation: a case report

**DOI:** 10.1186/s13256-018-1843-4

**Published:** 2018-10-20

**Authors:** Jason Hinton, Alberto Cerra-Franco, Kevin Shiue, Lindsey Shea, Vasantha Aaron, Geoffrey Billows, Ahmad Al-Hader, Tim Lautenschlaeger

**Affiliations:** 1 Department of Radiation Oncology, Indiana University School of Medicine, 535 Barnhill Drive, Cancer Care Pavilion 041, Indianapolis, IN 46202-5289 USA; 2 Department of Radiology, Indiana University School of Medicine, 550 North University Boulevard, Room 0663, Indianapolis, IN 46202 USA; 3 Department of Emergency Medicine, Indiana University School of Medicine, 720 Eskenazi Avenue, Fifth Third Bank Building 3rd Floor, Indianapolis, IN 46202 USA; 4 Department of Hematology and Oncology, Indiana University School of Medicine, 980 West Walnut Street R3 C312, Indianapolis, IN 46202 USA

**Keywords:** Radiation-induced SVC syndrome, SVC syndrome

## Abstract

**Background:**

The incidence of superior vena cava syndrome within the United States is roughly 15,000 cases per year. Superior vena cava syndrome is a potentially life-threatening medical condition; however, superior vena cava syndrome is not fatal in the majority of cases. Superior vena cava syndrome encompasses a collection of signs and symptoms resulting from obstruction of the superior vena cava, including swelling of the upper body of the head, neck, arms, and/or breast. It is also associated with cyanosis, plethora, and distended subcutaneous vessels. Lung cancer, including both non-small cell lung cancer and small cell lung cancer, is the most common extrinsic cause of superior vena cava syndrome. Intrinsic disruption of superior vena cava flow can also precipitate superior vena cava syndrome. This case report describes an unusual presentation and potential etiology of superior vena cava syndrome.

**Case presentation:**

Our patient was a 51-year-old black woman with locally advanced, stage IIIB non-small cell lung cancer who had no clinical symptoms of superior vena cava syndrome at the time of diagnosis. However, she did have radiographic evidence of superior vena cava stenosis caused by extrinsic compression from her large right hilar primary tumor. She was treated with definitive chemoradiation, receiving 60 Gy of external beam radiation therapy given concurrently with chemotherapy. Three months after completion of radiotherapy, she developed signs of superior vena cava syndrome, including breast and supraclavicular swelling. She had a chest computed tomography scan showing over 50% reduction in the size of a right hilar mass; however, she had continued radiographic stenosis of the superior vena cava. The distribution of stenosis appeared to be inferior to the caudal extent of pretreatment tumor volume. She had no other radiographic indications for superior vena cava syndrome.

**Conclusions:**

Generally, superior vena cava syndrome is the result of extrinsic compression of the superior vena cava by tumor. Our patient’s case represents the development of superior vena cava syndrome after an excellent response of tumor with near-complete tumor response. We suspect chemoradiation therapy as a potential etiology for the precipitation of the superior vena cava syndrome, which is currently not well reported in the medical literature.

## Background

Superior vena cava (SVC) syndrome encompasses a collection of signs and symptoms resulting from obstruction of the SVC, including swelling of the upper body of the head, neck, arms, and/or breast. It is also associated with cyanosis, plethora, and distended subcutaneous vessels. The edema that develops may cause functional deficits of the larynx or pharynx, contributing to cough, hoarseness, dyspnea, and dysphagia.

SVC syndrome historically was considered an oncologic emergency and was one of only a handful of emergent indications for palliative radiation therapy. The incidence of SVC syndrome within the United States is roughly 15,000 cases per year [1]. SVC syndrome is a potentially life-threatening medical condition; however, it is not fatal in the majority of cases [2]. A retrospective literature review including 1986 patients with SVC syndrome between 1934 and 1984 reported only 1 death that could be directly attributed to SVC obstruction [2].

SVC syndrome can be the result of either extrinsic compression or intrinsic disruption of venous blood flow. The pace of the onset of venous restriction is the main driver behind the development of SVC syndrome. Slowly developing SVC lesions can result in the development of collateral circulation through the inferior vena cava and the azygous vein that can mitigate or even eliminate the development of SVC syndrome.

Lung cancer, including both non-small cell lung cancer (NSCLC) and small cell lung cancer, is the most common extrinsic cause of SVC syndrome [1]. Two to four percent of all patients with lung cancer develop SVC syndrome at some point during their disease course [3–5]. SVC syndrome is more common in small cell lung cancer, occurring in approximately 10% of cases [6]. Other extrinsic etiologies of SVC syndrome include lymphoma and metastatic disease, as well as nonmalignant causes, including aortic aneurysm [7, 8]. Intrinsic causes include both superior and inferior vena cava stenosis, thrombosis, and use of intravenous catheters. Radiation has only briefly been described in the literature as an etiology of SVC syndrome [9–11].

Generally, SVC syndrome is the result of extrinsic compression of the SVC by tumor. Our patient’s case represents the development of SVC syndrome after an excellent near-complete tumor response. We suspect that chemoradiotherapy is a potential etiology for the precipitation of the SVC syndrome, which is currently not well reported in the medical literature. This case report adds to the medical literature by describing radiation as a potential cause of SVC syndrome.

## Case presentation

A 51-year-old black woman with an 18-pack-year smoking history presented to our institution with a 3-month history of a progressively productive cough unresponsive to antibiotics. In addition, she had dyspnea on exertion and a 25-pound weight loss. Her past medical history included a duodenal ulcer resulting in a perforation which required exploratory laparotomy 2 years prior to presentation. Other history included subarachnoid hemorrhage requiring craniotomy with hematoma evacuation roughly 20 years prior to presentation, as well as hypertension. Her family history included her mother being diagnosed with ovarian cancer at the age of 54. The patient is married and worked full-time at the front desk for the past 30 years for a shipping company. She reported alcohol intake of two drinks per occasion twice weekly. She denied the use of any recreational drugs. She denied any environmental exposures. Medications that the patient was receiving at the time of diagnosis included amlodipine and albuterol.

**Fig. 1:**
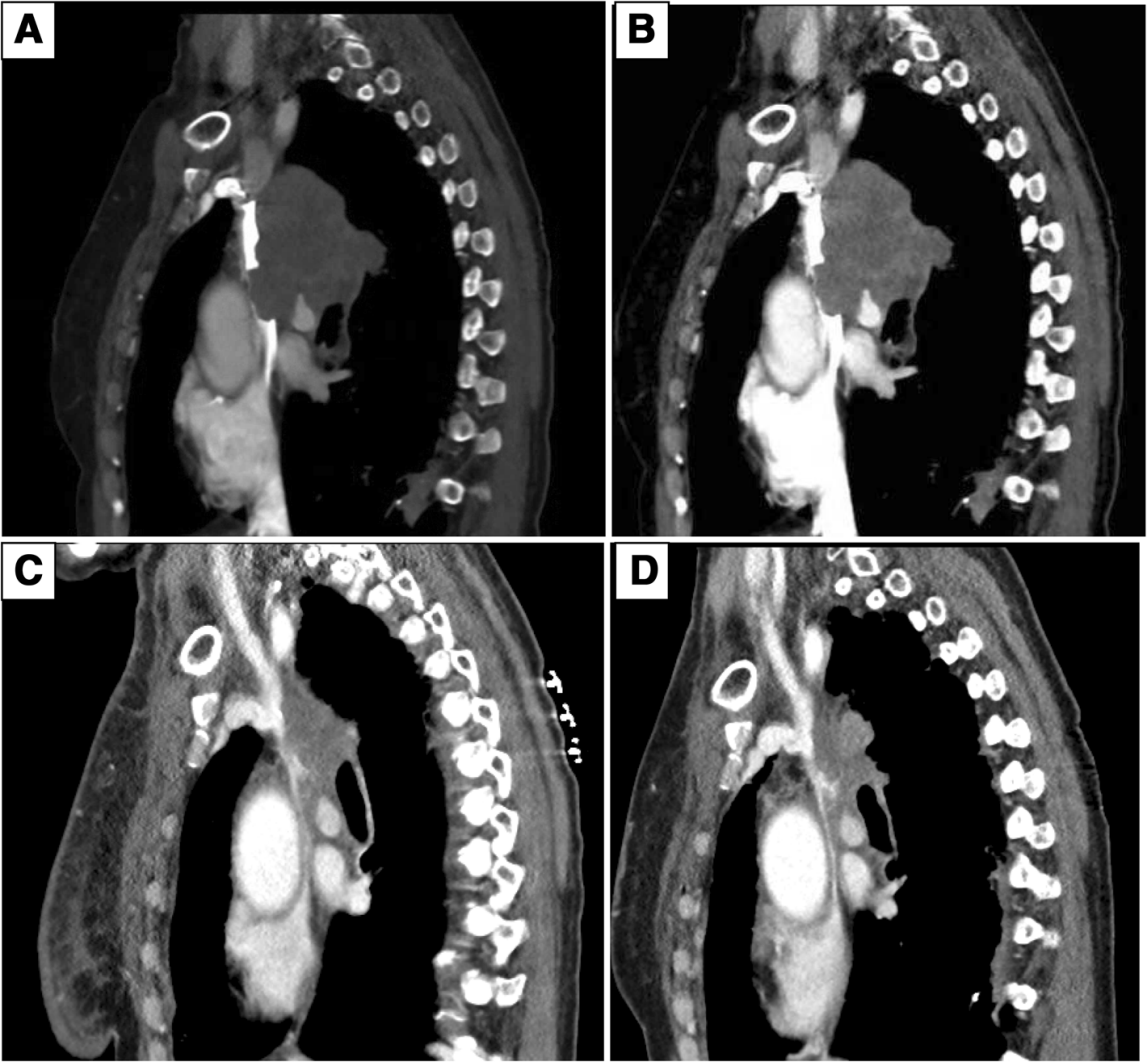
Superior vena cava (SVC) is patent pretreatment, as seen by contrast below the tumor in (**a**) and (**b**). The inferior portion of the SVC is narrowed 3 and 6 months posttreatment, as seen in (**c**) and (**d**), respectively

The patient underwent computed tomography (CT) of the chest, which revealed a 5.3 × 6-cm right hilar mass that was occluding the right upper lobe bronchus with narrowing of the SVC. The SVC remained radiographically patent (Fig. 1a, b). The patient’s vital signs included afebrile temperature of 37.0 °C, cardiac pulse of 100 beats per minute, and oxygen saturation of 96% on room air. Her physical examination at that time was without any clinical signs of venous congestion. She had no facial plethora and had flat neck veins and no signs of jugular vein distention. She had decreased breath sounds in the right upper and middle lung fields. The skin of the neck and breast was without any pitting or edema. Neurologically, the patient was fully functional with cranial nerves II–XII intact and 5/5 strength in the upper and lower extremities bilaterally. All laboratory test results, including complete blood count and comprehensive metabolic panel, were within normal limits.

**Fig. 2:**
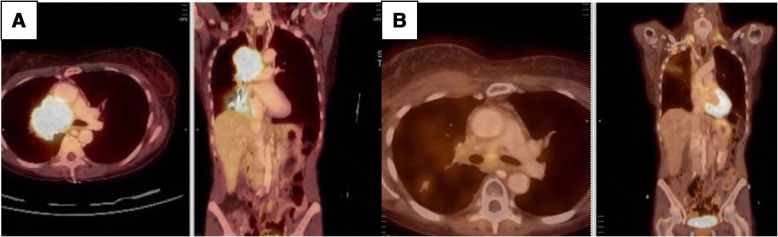
Positron emission tomography/computed tomography shows large right hilar mass pretreatment (**a**) and 3 months posttreatment (**b**)

Two weeks after the patient’s initial presentation, she was found to have bilateral distended venous jugular veins, with no facial, neck, or breast fullness, which was self-limiting and resolving prior to chemoradiation. She eventually underwent an endobronchial ultrasound with fine-needle aspiration of the right hilar mass along with the contralateral mediastinal station 4L lymph node demonstrating poorly differentiated NSCLC adenocarcinoma in both sites. She then underwent brain magnetic resonance imaging (MRI) and positron emission tomography (PET)/CT (Fig. 2a), which revealed no evidence of metastatic disease. She was diagnosed with T2bN3M0 stage IIIB lung adenocarcinoma according to American Joint Committee on Cancer 8th edition staging guidelines.

**Fig. 3:**
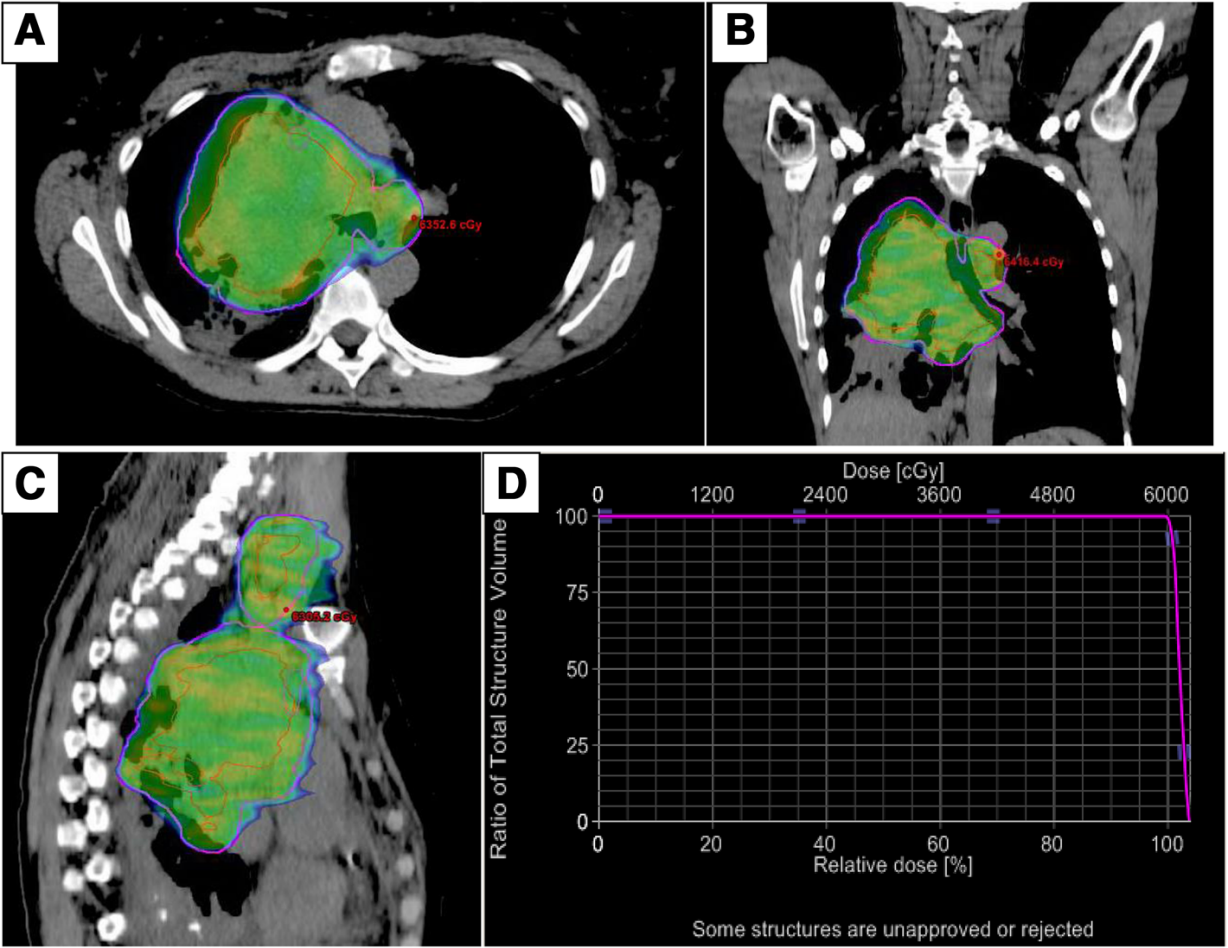
Radiation plan showing 95% isodose coverage (57 Gy). The entire superior vena cava (SVC) is within the planning target volume or high-dose region of radiotherapy. (**a**) Axial, (**b**) coronal, and (**c**) sagittal planning computed tomographic scans. **d** Dose-volume histogram analysis of SVC (magenta)

The patient proceeded with expedited radiation planning and treatment, given her radiographic evidence of extrinsic SVC narrowing and physical examination findings of mild and self-limiting jugular venous distention. She received a definitive radiation dose of 60 Gy in 30 fractions concurrent with chemotherapy (cisplatin and etoposide). The patient was simulated in the supine position in a whole-body Vac-Lok ™ (CIVCO Radiotherapy, Orange City, IA, USA) with arms above her head. A four-dimensional (4D) CT simulation was performed using a Philips Ingenuity CT simulation scanner (Philips, Cleveland, OH, USA) to acquire images for treatment planning and assessment of internal target motion. Treatment planning was performed using an Eclipse® treatment planning system (Varian Medical Systems, Pao Alto, CA, USA), and treatment was delivered using a TrueBeam® radiotherapy system (Varian Medical Systems) with two volumetric modulated arc therapy arcs using 6-MV photons. Gross tumor volume (GTV) was contoured on 4D CT images in different phases of the respiratory cycle. An internal target volume was created by the summation of GTV volumes of the different respiratory phases. A 5-mm expansion was used to create the clinical target volume and planning target volume (PTV), respectively. Ninety-five percent of the PTV received at least 57 Gy or 95% of the prescribed dose (Fig. 3). Cycle 1 of cisplatin (50 mg/m^2^ on days 1, 8, 29, and 36) plus etoposide (50 mg/m^2^ daily on days 1 to 5 and days 29 to 33) started 2 weeks after initiation of radiation therapy. One additional cycle was given after completion of radiation for a total of two cycles of chemotherapy received. The patient did experience some breast swelling and pain that was seen with day 10 of chemotherapy. The event was suspected to be a consequence of the excess intravenous fluids (3 L) coadministered with each infusion of cisplatin. This event was in the setting of the previous stenosis of the SVC resulting from extrinsic compression from the patient’s large right hilar mass. This swelling resolved within 1 week without intervention. The patient also developed esophagitis requiring temporary gastric tube placement necessitating a 5-day hospitalization.

The patient returned to the radiation oncology department for a 3-month posttreatment follow-up visit, for which imaging was ordered. The patient reported that for the past 2 weeks she had developed swelling and pain in her right breast and right supraclavicular region, which were new for her. She also complained of worsening dyspnea on exertion, a 16-pound weight gain over the past 3 months, and intermittent headaches. She denied any facial fullness, orthopnea, or dysphagia. Examination revealed that her vital signs were within normal limits; however, she had obvious compressible swelling of the right supraclavicular region and fullness within the right breast. Chest CT performed 2 days prior to the follow-up visit revealed a remarkable reduction in size by approximately 50% of the treated right hilar mass (Fig. 1c). However, the SVC was significantly narrowed to completely occluded radiographically, despite being narrowed but patent before treatment. The patient was sent to the emergency department for further evaluation and management. Interventional radiology was consulted, and it was concluded that the patient’s SVC syndrome was likely chronic based on the presence of significant collaterals. No acute intervention was recommended, and she was discharged to home uneventfully.

The patient did have initial radiographic evidence of stenosis of the SVC; however, her initial stenosis did not produce SVC syndrome. The patient did respond very well to treatment, because she had marked reduction of the tumor volume and decrease in the extent of extrinsic compression on the SVC. It was peculiar that she developed SVC syndrome despite her significant treatment response.

We also contemplated intrinsic etiologies for SVC syndrome, including thrombus formation, but this consideration was not confirmed on radiographs. Interventional radiology was consulted and had reviewed her films and did not think thrombus was a likely scenario. There was a concern for recurrent or persistent microscopic disease within the region of the SVC, but there was no fludeoxyglucose avidity in the region on posttreatment PET (Fig. 2b). The development of an adequate collateral circulation system did indicate the chronicity of venous congestion. The differential diagnosis also included the possibility that the continued stenosis of the SVC was a direct effect of treatment (chemoradiation) because the SVC resided within the PTV receiving the full radiation dose. The SVC was contoured, and dosimetric parameters were as follows: Minimum dose to SVC was 59.74 Gy with maximum dose to SVC 62.36 Gy, and 95% of the SVC received at least 60.35 Gy (Fig. 3).

The patient was presented at the multidisciplinary thoracic oncology tumor board, and observation was recommended on the basis of absence of tumor progression and presence of an adequate collateral venous system. Her supraclavicular and breast swelling was self-limited, resolving within 2 weeks after her presentation. Six months after completion of radiation therapy, she underwent PET/CT indicating recurrent thoracic disease and had a fine-needle aspiration biopsy confirming metastatic, poorly differentiated adenocarcinoma in a station 4R lymph node. She was placed on the PD-1 (programmed cell death protein 1) inhibitor nivolumab. Brain MRI was performed for workup, revealing two ring-enhancing lesions in the left frontal and right cerebellum, the largest of which was 2.2 × 1.8-cm in the left frontal lobe. Gamma Knife® (Elekta, Stockholm, Sweden) stereotactic radiosurgery was performed on the two lesions. One year after completion of definitive thoracic radiotherapy, the patient was found to have a new metastatic left parieto-occipital brain lesion that was being worked up at the time of this report. She has not had redevelopment of SVC syndrome.

## Discussion

SVC syndrome generally is caused by extrinsic compression of the flexible SVC by tumor. The unique characteristic of this case is that the SVC syndrome developed with decreasing extrinsic compression. Radiation is suspected as the direct cause of SVC syndrome in our patient, which adds to the paucity of data portraying radiation as an etiology of SVC syndrome.

Radiation-induced injury to blood vessels was discovered by pathologists soon after the invention of x-rays [12]. However, radiation damage to veins has not been evaluated as well as radiation damage to the arterial system [13, 14]. Radiation-related pathologic changes within veins include intimal proliferation and formation of fibrous plaques [12]. Several case reports describe radiation-induced SVC syndrome. These case reports all describe radiation-induced fibrosis and SVC syndrome that developed years after completion of radiation therapy. Lee *et al.* described a case a 59-year-old man who received adjuvant radiation therapy for primary germinal cell tumor of the mediastinum [6]. The patient underwent resection of a mediastinal mass followed by radiation therapy to a total dose of 32 Gy given in 2-Gy fractions. His treatment was performed 20 years prior to the development of SVC syndrome, which manifested as a headache with facial and neck edema. There was radiographic evidence of complete occlusion of the SVC. Venous decompression was achieved by left internal jugular anastomosis to the right atrium. During surgical resection, the proximal portion of SVC, right brachiocephalic vein, and innominate vein appeared to be fibrotic, with biopsy confirming fibrosis without recurrence of the tumor. Mehta *et al.* described a patient who received definitive radiotherapy in Cuba for Hodgkin’s lymphoma 50 years prior to her presentation of SVC syndrome [9]. No other cause for SVC was identified, and the radiation dose is unknown. Van Putten *et al*. also reported cases of two patients with suspected radiation-induced SVC syndrome [11]. The first patient was a 47-year-old man who received 60 Gy to mediastinal paratracheal nodes adjacent to the SVC in the treatment of recurrent lung cancer 2 years after pneumonectomy. Five years after radiation therapy, the patient developed SVC syndrome manifested as facial and neck swelling without CT or PET evidence of recurrence of the mediastinal mass. The SVC syndrome resolved with stent placement. The second patient was a 36-year-old man who developed solitary metastatic disease to a right-sided cervical lymph node receiving 50 Gy in 2-Gy fractions to the bilateral neck in the cervical and supraclavicular regions along with a 20-Gy boost to the gross tumor with electron beams. Seven years after radiotherapy, he developed right arm swelling. Venography confirmed no flow through the brachiocephalic vein, and the patient ultimately underwent mediastinoscopy and sternotomy, confirming fibrosis without any evidence of malignancy. PET also confirmed no evidence of recurrent disease.

## Conclusions

Our patient’s case differs from the previously mentioned cases in that the formation of the SVC syndrome occurred just 3 months after the completion of radiotherapy, much earlier than previously reported. Our patient did have radiographic, but not clinical, evidence of SVC stenosis at the time of diagnosis, which was due to her very large hilar mass with extrinsic compression of the SVC. The unique part of our patient’s case is the fact that development of SVC syndrome coincided with remarkable reduction in the size of her tumor and presumed lessening of the extrinsic compression. The majority of the patient’s SVC was within the full-dose region of radiotherapy. It is possible that the development of the SVC syndrome was a chemoradiation-related event, but it is not uncommon for the SVC to get the full dose of radiation in the treatment of thoracic malignancies. In addition, the location of the SVC stenosis changed when we compared the pre- and the posttreatment radiologic scans. It appears that up to an 8-cm segment of SVC was narrowed after treatment, some of which was below the initial area of compression but within the high-dose radiation area. Furthermore, it was considered that the SVC syndrome could be related to microscopic extension of the tumor beyond what could be identified on imaging studies. The possibility of microscopic tumor extension more caudally in the SVC (i.e., the region that was found to be stenotic at follow-up) should be considered, even though there was no macroscopic tumor in that area seen on CT scans. In conclusion, we believe that our patient’s case displays a variety of potential etiologies for SVC syndrome, including radiation therapy.

## Abbreviations

4D: Four-dimensional
CT: Computed tomography
GTV: Gross tumor volume
MRI: Magnetic resonance imaging
NSCLC: Non-small cell lung cancer
PD-1: Programmed cell death protein 1
PET: Positron emission tomography
PTV: Planning target volume
SVC: Superior vena cava
